# Small nucleolar RNA signatures of lung tumor-initiating cells

**DOI:** 10.1186/1476-4598-13-104

**Published:** 2014-05-06

**Authors:** Kaiissar Mannoor, Jun Shen, Jipei Liao, Zhenqiu Liu, Feng Jiang

**Affiliations:** 1 Departments of Pathology, University of Maryland School of Medicine, Baltimore, MD, USA; 2 Division of Biostatistics of The University of Maryland Greenebaum Cancer Center, Baltimore, MD, USA

## Abstract

**Background:**

Non-small cell lung cancer (NSCLC) is the number one cancer killer. Tumor-initiating cells (TICs) are responsible for tumor progression and recurrence. Emerging evidences suggest that small nucleolar RNAs (snoRNAs) play malfunctioning roles in lung tumorigenesis. This study aims to determine if snoRNAs have important function in lung TICs by: 1) profiling and comparing snoRNA expression patterns in lung ALDH1+/- cells of 28 primary NSCLC tissues to identify new signatures of TICs; 2) determining prognostic significance of the snoRNA signatures by analyzing the expression in 82 NSCLC tissues with different stages and histological types using quantitative PCR; 3) functionally investigating if the snoRNAs contribute to stemness of lung TICs using in vitro and in vivo assays.

**Results:**

Twenty-two snoRNAs were identified whose changes were specific to the TICs. The expression of two snoRNAs (snoRA3 and snoRA42) was inversely associated with survival of NSCLC patients (P = 0.002, p = 0.001, respectively). Functional analysis indicated that snoRA42 was upregulated in CD133+ cells isolated from NSCLC cell lines compared with the CD133- counterparts. snoRA42 knockdown reduced the proliferation and self-renewal of TICs in vitro. However, ectopic expression of snoRA42 in non-TICs enhanced the potentials of cell proliferation and self-renewal. snoRA42 expression was associated with expression of stem cell-core transcription factors in lung TICs. Blocking snoRA42 expression in TIC xenografts decreased tumorigenesis in mice.

**Conclusions:**

The snoRNA signatures of lung TICs provide potential biomarkers for predicting outcome of NSCLC. snoRA42 is one of the important snoRNAs in regulating features of lung TICs, and thus contributes to lung tumorigenesis.

## Background

Non-small cell lung cancer (NSCLC) is the leading cause of cancer death for men and women worldwide. With availability of more sensitive radiological imaging studies, more NSCLC patients will be diagnosed while the disease is still at early stage [1,2]. The standard of care for NSCLC is surgery, often followed by chemotherapy [1]. However, approximately 84% of those diagnosed with lung cancer will die within five years [1]. Furthermore, the current chemotherapies often have toxicity in normal host tissues, whereas tumor cells rapidly develop resistance to anticancer agents. The developments of biomarkers for identifying NSCLC patients at high risk of recurrence after surgery, and therapeutic targets for safe and effective treatment of lung cancer are clinically important.

The existence of tumor-initiating cells (TICs), also known as cancer stem cells, could explain why the current chemotherapies cannot consistently eradicate tumors, because the therapies only target the bulk of tumor cells and are unable to eliminate TICs [3]. Furthermore, residual lung TICs may regenerate a cancer cell population, leading to tumor relapse after therapy. TICs have been identified in lung cancer by using several approaches such as CD133, a cell surface marker [4]. We have recently characterized ALDH1+ cancer cells are TICS, as the ALDH1+ cancer cells have extensive self-renewal, proliferative, and *in vivo* tumorigenic potentials [5-7]. The analysis of molecular aberrations that characterize TICs would deep our understanding of lung tumor biology. Furthermore, the molecular changes could be developed as a new diagnostic system for monitoring outcome of NSCLC. In addition, the TIC-related molecular changes may enable the development of specific agents for eradicating the tumor-maintaining cells, and thus provide efficient therapeutic approaches for lung cancer.

Non-coding RNAs (ncRNAs) are functional transcripts that do not code for proteins, however, play a crucial role in regulating gene expression [8]. ncRNAs include small nucleolar RNAs (snoRNAs), microRNAs (miRNAs), short interfering RNAs (siRNAs), piwi-associated RNAs, small Cajal body-specific RNAs (scaRNAs), snRNAs (small nuclear RNAs), and long ncRNAs [8]. Of the small ncRNAs, miRNAs have extensively been studied in carcinogenesis [9,10]. Dysregulation of some miRNAs is associated with features of TICs. For example, elevated miR-181 clusters were identified as vital regulators in hepatic TICs [11]. Furthermore, up-regulation of miR-199b-5p impaired the development of TICs of medulloblastoma though repression of HES1 [12]. In addition, the restoration of miR-34 expression suppressed the self-renewal of pancreatic TICs [13].

Recently, new and unexpected functions of other types of small ncRNAs have been discovered, revealing that the molecules have highly diverse roles and are actively involved in the processes of carcinogenesis than previously thought [10]. In particular, several studies including our own data suggest that snoRNAs exhibit differential expression patterns in lung tumor and have capability to affect cell transformation, tumorigenesis, and metastasis of NSCLC [10,14,15]. snoRNAs range between 60–150 nucleotides in length [16]. There are two classes of snoRNAs: box C/D snoRNAs (snoRDs) and box H/ACA snoRNAs (snoRAs) [16]. snoRDs serve as guides for the 2′-O-ribose methylation of rRNAs or snRNAs, whereas snoRAs are guides for the isomerization of uridine residues into pseudouridine [17]. Accumulated evidence suggests that snoRNAs can target other RNAs including snRNAs and messenger RNAs [17]. For instance, HBII-52 regulates alternative splicing of 5-HT2CR by binding to a silencing element in exon Vb [18,19]. Therefore, profiling the expression patterns of snoRNAs in lung TICs may help comprehend the functional repercussions of the ncRNAs in the development and progression of NSCLC, and hence provide diagnostic biomarkers and therapeutic targets for the disease.

We propose to determine if aberrant snoRNAs have important function in lung TICs by: 1) profiling and comparing snoRNA expression patterns in lung ALDH1+/- cells of primary NSCLC tissues to identify signatures of TICs; 2) determining prognostic significance of the signatures by analyzing the expression in NSCLC tissues; 3) functionally investigating if the snoRNA signatures may contribute to stemness of lung TICs.

## Results

### Identifying snoRNA signatures of lung TICs

**Figure 1:**
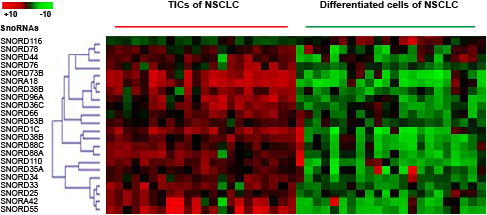
**1snoRNAs differentially expressed in TICs (ALDH1+ cells) versus non-TICs (ALDH1- cells) isolated from 22 non-small cell lung cancer (NSCLC) tumor tissues.** Hierarchical clustering of 22 snoRNAs with a significantly different expression (p < 0.01) in ALDH1+ cells versus ALDH1- cells isolated from surgical NSCLC tumor tissues. Rows represent individual genes; columns represent individual samples.

To identify snoRNA signatures of lung TICs, we first used Aldefluor assay and fluorescence-activated cell sorting to isolate ALDH1+ and ALDH1-cancer cells from 28 primary NSCLC tumor tissues. The NSCLCs comprised 15 adenocarcinoma (ACs) and 13 squamous cell carcinomas (SCCs). Twenty-two of 28 NSCLC tissues yielded ALDH1+ cells with an average of 2.2% (±0.66), ranging from 0.7% to 3.2% of gated cells. We then used the GeneChipR Array to analyze snoRNA expression on the ALDH1 + cells and ALDH- cells of the lung tumors. The GeneChipR Arrays contained probes for 352 human mature snoRNAs. When P value <0.01 was used as a cutoff, 22 snoRNAs displayed a differential expression level with ≥ 3.0 fold-change in ALDH1+ cancer cells compared with the corresponding ALDH1- cancer cells (Figure 1 and Table 1). Of the 22 snoRNAs, 21 snoRNAs were overexpressed and one snoRNA was underexpressed in ALDH1+ cancer cells compared with the corresponding ALDH1- cancer cells. The 22 snoRNAs provide new signatures of lung TICs.

**Table 1:** Up-regulated and down-regulated snoRNAs in TICs vs. non-TICs

**Name of snoRNA**	**Fold-change (TICs/non-TICs)**	**p. Value**
SNORA42	4.383	0.00117
SNORA3	4.144	0.00010
SNORA61	3.893	0.00010
SNORD14E	3.733	0.00021
SNORA62	3.600	0.00035
SNORD78	3.538	0.00874
SNORD44	3.505	0.00084
SNORD76	3.503	0.00001
SNORD73B	3.468	0.00006
SNORA18	3.456	0.00199
SNORD38B	3.430	0.00066
SNORD96A	3.405	0.00043
SNORD36C	3.387	0.00079
SNORD66	3.367	0.00005
SNORD83B	3.362	0.00029
SNORD88A	3.326	0.00003
SNORD110	3.303	0.00058
SNORD34	3.206	0.00105
SNORD33	3.169	0.00031
SNORD1C	3.055	0.00005
SNORD55	3.005	0.00130
SNORD116-26	-3.052	0.00505

TICs, tumor-initiating cells; non-TICs, non-tumor-initiating cells.

TICs were ALDH1 cells purified by Aldefluor assay.

### Clinical significance of the lung TIC-snoRNA signatures

**Figure 2:**
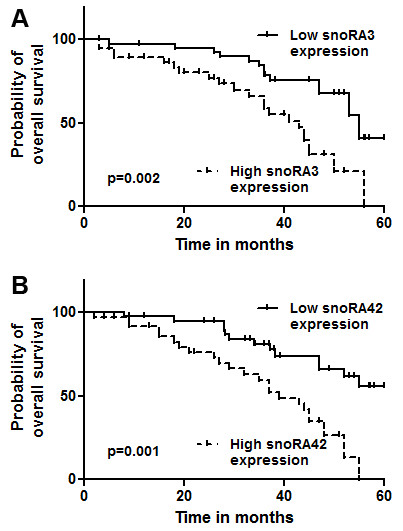
**Expression of snoRA3 and snoRA42 in lung tumor tissues is inversely associated with survival of non-small cell lung cancer (NSCLC) patients. (A)** Probability of overall survival by levels of snoRA3 expression in NSCLC. **(B)** Probability of overall survival by levels of snoRA42 expression in NSCLC.

snoRA3 and snoRA43 displayed the highest expression levels in TICs compared with non-TICs, and therefore were chosen in the present study to be evaluated for the clinical significance in 82 NSCLC tissues by quantitative reverse transcriptase PCR (qRT-PCR) assay. snoRA3 and snoRA43 had <35 threshold cycle (Ct) value in all the tissue specimens. Univariate Cox regression analysis showed that expression of the two snoRNAs, age, and stage were significantly associated with survival of the patients (All P < 0.05) (Additional file 1: Table S2). However, there was no statistical significant correlations between either race, or sex, or tumor histological type, or smoking history and survival of the patients (All P > 0.05). Furthermore, the Kaplan-Meier Curve analysis suggested that the NSCLC patients with a high expression level of the snoRNAs had a poor survival compared with the patients who had a lower level of the snoRNAs in the tumor tissues (P = 002 and P = 001, respectively) (Figure 2). In addition, multivariate Cox proportional-hazards model analyses indicated that the two snoRNAs were statistically significant after adjusting patient age and tumor stage, suggesting their independent prognostic value from the clinicopathologic features (P = 0.003 and P = 0.002, respectively) (Additional file 1: Table S1). Therefore, the assessment of the two TIC-snoRNA signatures might provide a potential approach for predicting outcome of NSCLC.

### Dysregulation of snoRA42 is associated with properties of TICs

#### snoRA42 is highly expressed in CD133+ cells isolated from NSCLC cell lines

**Figure 3:**
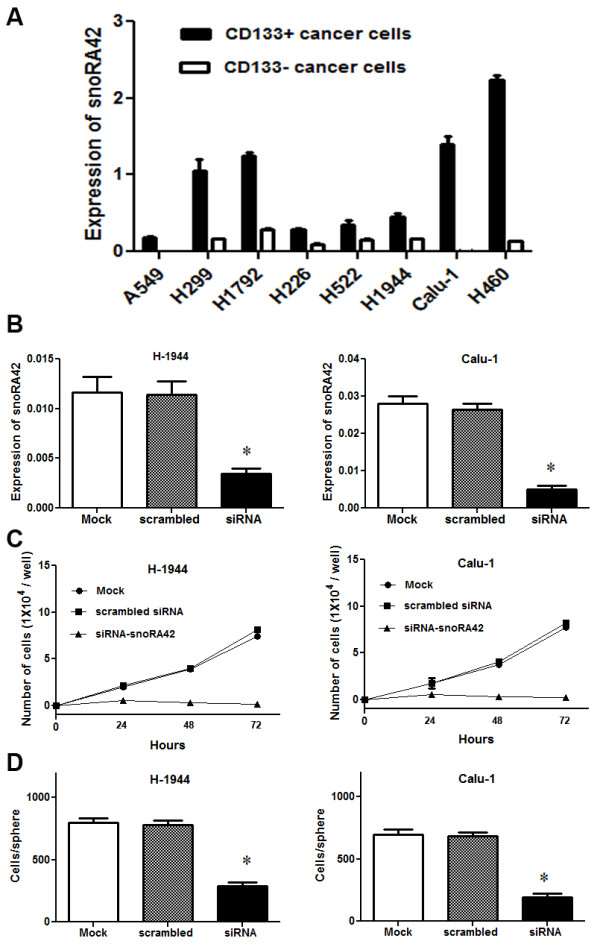
**siRNA-mediated snoRA42 suppression inhibits proliferation and self-renewal of CD133+ cells isolated from NSCLC cell lines. (A)** There was a high level of expression of snoRA42 in CD133+ cells versus CD133- cells of a panel of NSCLC cells determined by qPCR analysis. **(B)** siRNA-snoRA42 efficiently reduced snoRA42 expression in CD133+ cells isolated from NSCLC cell lines. qPCR analysis was performed to determine snoRA42 expression in CD133+ cells treated with mock transfection, scrambled siRNA, or siRNA-snoRA42. **(C)** downregulation of snoRA42 decreased cell growth of CD133+ cells isolated from NSCLC cell lines. The time-kinetics of cell proliferation rate of CD133+ cancer cell with mock transfection, scrambled siRNA transfection, or siRNA-snoRA42 transfection was determined. Cell proliferation rate after snoRA42 knockdown in CD133+ cancer cells was significantly reduced after 24 hours. **(D)** snoRA42 knockdown reduced the percentage of mammospheres of CD133+ cells. The experiments were performed in all the NSCLC cell lines listed in Methods and had similar results. The number of cells used in each experiment can be found in Methods. The figure only shows results from H-1944- and Calu-1-derived CD133+ cells. *P values <0.05.

We used snoRA42 as an example to investigate whether the TIC-snoRNA signatures could have important function in TICs. Because the snoRNA signatures were defined from ALDH1+ cancer cells and CD133 is another important marker for lung TICs, we used CD133+ and CD133- cells that were isolated from a panel of NSCLC cell lines to confirm if snoRA42 was specifically dysregulated in TICs. As shown in Figure 3A, snoRA42 expression increased by at least 2.5-fold in CD133+ cancer cells compared with the CD133- counterparts, supporting that the snoRNA signature was enriched in lung TICs.

#### Downregulation of snoRA42 inhibits proliferation and self-renewal of lung TICs

We explored whether snoRA42 downregulation could inhibit cell growth and proliferation of lung TICs by transfecting CD133+ cells isolated from NSCLC cell lines with snoRA42-siRNA. snoRA42-siRNA produced more than 75% reduction of snoRA42 expression in the CD133+ cells compared with mock-transfected CD133+ cells or CD133+ cells transfected with the scrambled siRNA (Figure 3B). The results suggested that the snoRA42-siRNA could block snoRA42 expression in TICs. Furthermore, there was a significant decrease of numbers of CD133+ cancer cells transfected with snoRA42-siRNA compared with those with scrambled siRNA or mock transfection (Figure 3C). The inhibition of snoRA42 silencing on cell growth of TICs was confirmed by methylthiazol tetrazolium (MTT) assay. The findings suggest that snoRA42 knockdown could have a significant inhibition on growth and proliferation of lung TICs. In addition, CD133+ cancer cells transfected with snoRA42-siRNA gave rise to significantly fewer mammospheres (Figure 3D) compared with CD133 + cancer cells treated with controls, implying the downregulation of snoRA42 decreased the capacity of mammosphere formation of TICs. Therefore, reduced snoRA42 expression in TICs could inhibit the self-renewing capacity of lung TICs.

#### Downregulation of snoRA42 reduces in vitro tumorigenesis of CD133+ cells isolated from NSCLC cell lines

**Figure 4:**
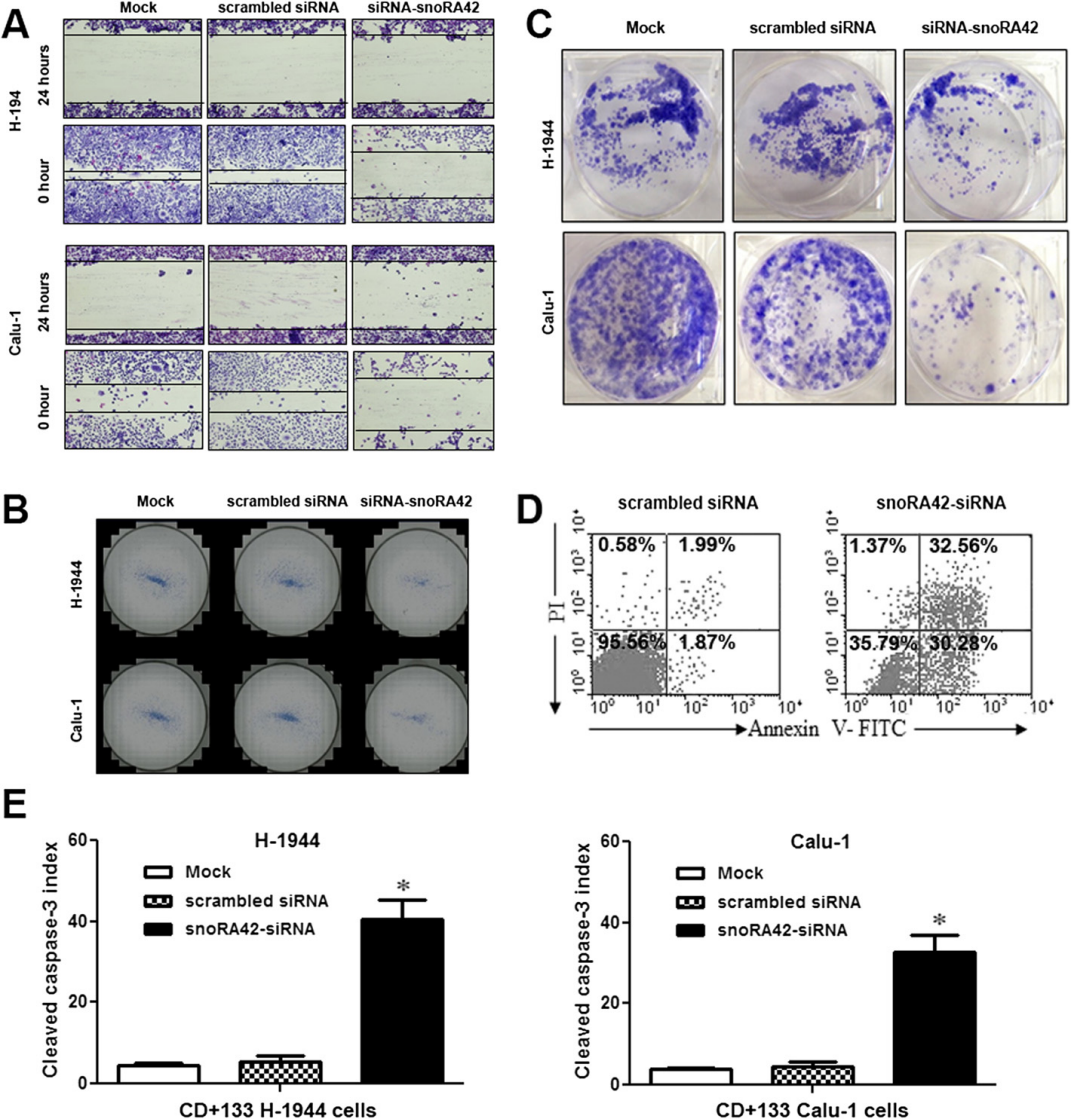
**Downregulation of snoRA42 reduces in vitro tumorigenesis of CD133+ cells isolated from NSCLC cells. (A)** snoRA42 knockdown inhibited migration rate of H-1944- and Calu-1-derived CD133+ cells. A wound scratch assay was used to compare the migration rate of cells treated with mock transfection, scrambled siRNA, or siRNA-snoRA42. The cells treated with siRNA-snoRA42 had a significantly higher level of migration rate compared with the cells treated with mock transfection or scrambled siRNA. **(B)** downregulation of snoRA42 moderated matrigel barrier invasion of H-1944- and Calu-1-derived CD133+ cells. Matrigel barrier invasion capability was compared among three different groups of CD133+ cells treated with mock, scrambled siRNA, or snoRA42-siRNA. **(C)** snoRA42 knockdown significantly inhibited colony formation capability of H-1944- and Calu-1-derived CD133+ cells. **(D)** snoRA42 knockdown induced apoptosis. Apoptosis of H-1944-derived CD133+ cells treated with scrambled siRNA and snoRA42-siRNA was compared by staining the cells with Annexin V/PI (propidium iodide) followed by flowcytometric analysis. The numbers indicate percentages of non-stained cells (Annexin V - PI-), early apoptotic cells (Annexin V + PI-) and late apoptotic or necrotic cells (Annexin V + PI+). **(E)**. snoRA42A knockdown caused activation of caspase 3 in H-1944- and Calu-1-derived CD133+ cells. ELISA assay was used to determine cleaved caspase 3 protein in CD133+ cancer cells treated with mock transfection, scrambled siRNA, or siRNA-snoRA42. CD133+ cells showed a higher level of caspase 3 activation compared with CD133- cells after snoRA42 knockdown. *P values <0.05. The number of cells used in each experiment can be found in Methods.

CD133+ cancer cells have been reported to exhibit highly tumorigenic potential in terms of migration and invasion [4,20,21]. We therefore investigated whether snoRA42 could play a role in tumorigenesis of CD133+ cells isolated from NSCLC cell lines. As shown in Figure 4A, the spaces between the two edges of wound scratches were more spacious in siRNA-snoRA42- treated CD133+ cancer cells compared with scrambled siRNA- or mock-treated CD133+ cancer cells. Knockdown of snoRA42 inhibited the adhesion properties of CD133+ cancer cells, as manifested by lower density of cells 24 h post treatment than cell density before wound assay was started. Furthermore, there were significantly lower numbers of siRNA-snoRA42-treated CD133+ cells migrating across matrigel membrane compared with CD133+ cells treated with scrambled siRNA or mock transfection (Figure 4B). The findings suggested that snoRA42 knockdown could reduce migratory and invasive capabilities of lung TICs. In addition, we investigated whether siRNA-mediated suppression of snoRA42 could influence anchorage-independent long-term differentiation capacity of CD133+ cells by using soft agar colony formation assay. After four weeks, siRNA-snoRA42 resulted in a significant reduction of colony formation in terms of size and number of CD133+ cancer cells compared to CD133+ cancer cells treated with controls (Figure 4C). The results implied that snoRA42 suppression could reduce the *in vitro* tumorigenesis of CD133+ cells isolated from NSCLC cell lines.

#### snoRA42 knockdown induces caspase 3-dependent apoptosis

To investigate possible mechanism underlying the inhibition of *in vitro* tumorigenicity of TICs, we first evaluated whether there was apoptosis in CD133+ cancer cells treated with snoRA42-siRNA. As shown in Figure 4D, percentages of apoptotic cells were significantly increased upon snoRA42 knockdown compared with scrambled siRNA- and mock-treated CD133+ cancer cells. To confirm the finding, we applied enzyme-linked immunosorbent assay (ELISA) to analyze cell lysate for determining change of caspase-3. As shown in Figure 4E, cleaved caspase-3 was activated prominently in CD133+ cells treated with snoRA42-siRNA. Therefore, snoRA42 knockdown could trigger apoptosis in CD133+ cells isolated from NSCLC cell lines. The inhibition of cell proliferation and self-renewal of lung TICs by snoRA42 suppression may be achieved at least partially through inducing apoptosis.

#### Upregulation of snoRA42 increases in vitro tumorigenicity of TICs

**Figure 5:**
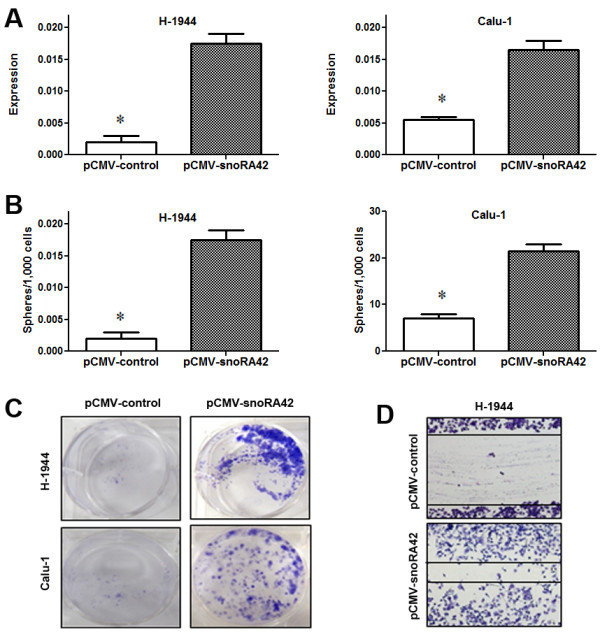
**Upregulation of snoRA42 in CD133- cells isolated from NSCLC cell lines increases the in vitro tumorigenicity. (A)** H-1944- and Calu-1-derived CD133- cells transduced with pCMV-snoRA42 displayed an elevated level of snoRA42 expression. **(B)** Effect of snoRA42 overexpression on sphere formation of H-1944- and Calu-1-derived CD133- cells. H-1944- and Calu-1-derived CD133- cells transduced with pCMV-snoRA42 had a higher growth rate of spheres compared with CD133- cells transduced with vector. **(C)** Effect of enforced expression of snoRA42 in H-1944- and Calu-1-derived CD133- cells on colony formation in soft agar. pCMV-control transduced and pCMV-snoRA42-transduced CD133- cells were allowed to differentiate for four weeks in soft agar, respectively. Forced expression of snoRA42 significantly increased colony formation capability of CD133- cells. **(D)** Ectopic expression of snoRA42 improved migration of H-1944-derived CD133- cells. A wound scratch assay was used to measure migration rate of cells transduced with pCMV-control and pCMV-snoRA42 24 h post treatment. Forced snoRA42 expression in CD133- cells caused a significant increase in migration capability of CD133- cells. *P values <0.05. The number of cells used in each experiment can be found in Methods.

To further determine the role of snoRA42 on stemness properties of TICs, we forced expression of snoRA42 in non-TICs by transducing CD133-cells with a vector of pCMV expressing snoRA42 (pCMV-snoRA42). As shown in Figure 5, CD133-cancer cells with pCMV-snoRA42 displayed a higher expression level of snoRA42 compared with CD133-cancer cells treated with pCMV control (Figure 5A). As a result, CD133-cancer cells with pCMV-snoRA42 formed bigger spheres in terms of number (Figure 5B) and size compared with CD133- cancer cells carrying pCMV control. Furthermore, cell number of the pCMV-snoRA42-transducted CD133- cancer cells considerably increased compared with that of CD133-cancer cells with pCMV control. In addition, there were significantly greater numbers of colonies of CD133-cancer cells with pCMV-snoRA42 compared to CD133-cancer cells with pCMV control (Figure 5C). Moreover, wound healing and matrigel transmigration assays showed that migration rate of CD133-cancer cells with pCMV-snoRA42 was higher compared with CD133-cancer cells with pCMV control (Figure 5D). Altogether, the gain-of-function experiments indicate that upregulation of snoRA42 would enhance the *in vivo* tumorigenicity of CD133- cancer cells isolated from NSCLC cells.

#### snoRA42 knockdown inhibits in vivo tumorigenesis of CD133+ cells isolated from NSCLC cell lines

**Figure 6:**
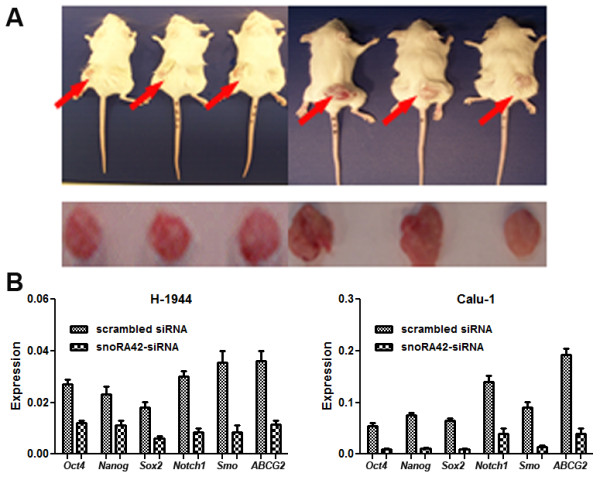
**Effect of snoRA42 suppression on tumor formation of CD133+ cells isolated from NSCLC cell lines and expressions of stem cell-associated genes. (A)** After six weeks, 5 X 10^5^ Calu-1-derived CD133+ cells transfected with scrambled siRNA were able to generate tumor xenografts (red arrow) in all six mice. However, 5 X 105 Calu-1-derived CD133+ cells transfected with snoRA42-siRNA could not produce tumor in any mice. **(B)** Knockdown of snoRA42 using snoRA42-siRNA in H-1944- and Calu-1-derived CD133+ cells resulted in a significant reduction in transcriptional activity of stem-associated genes determined by qRT-PCR.

To examine whether snoRA42 knockdown was associated with repression of *in vivo* tumorigenicity, we subcutaneously inoculated CD133+ Calu-1 NSCLC cells treated with either snoRA42-siRNA or scrambled siRNA into the flanks of mice (5 × 10^5^ cells/mouse). As shown in Figure 6, tumor appeared in 100% mice that received CD133+ Calu-1 NSCLC cells with scrambled siRNA control. In contrast, CD133+ Calu-1 NSCLC cells with snoRA42-siRNA were not capable of generating tumor in any of the mice injected. These results are consistent with those created from the above *in vitro* studies, and further support that snoRA42 is associated with tumorigenicity of lung TICs.

#### Dysregulation of snoRA42 is associated with expressions of stem cell-associated genes

To determine if snoRA42 suppression could affect expression of core stem cell transcription factors in CD133+ cells isolated from a panel of NSCLC cell lines, we quantified expression levels of stem cell-associated genes by qRT-PCR. siRNA-mediated knockdown of snoRA42 in CD133+ cells led to a significant decrease in transcript levels of the genes, including *Oct4, Nanog, Sox2, Notch1, Smo,* and *ABCG2* compared to scrambled siRNA-treated CD133+ cancer cells (Figure 6). Furthermore, to examine if enforced expression of snoRA42 could have an opposite effects to that of snoRA42 knockdown, we transduced CD133- cancer cells with pCMV-snoRA42. Although the expression levels of different stem cell-associated genes in snoRA42-transduced CD133- cancer cells could not reach to that in CD133+ cancer cells, transcriptional level of the stem cell-associated genes in CD133- cancer cells with pCMV-snoRA42 significantly increased compared to CD133- cancer cells with empty vector. Altogether, snoRA42 is associated with expression of the stem cell-core transcription factors in lung TICs.

## Discussion

Cancer arises from a tumorigenic subpopulation of TICs. The identification of molecular signatures of lung TICs provide a key standpoint for better understanding tumorigenesis and developing prognostic biomarkers and targeted therapy [22]. For instance, methylation of TIC-associated Wnt target genes were identified in colorectal tumors and showed the potential for predicting a poor prognosis of colon cancer patients [23]. Yet few molecular signatures of TICs in lung cancer have been identified. By conducting transcriptomic profiling ALDH1+/-NSCLC cells of primary tumor tissues, here we successfully identify 22 novel snoRNA signatures of lung TICs.

Although only two (snoRA3 and snoRNA42) of 22 snoRNAs are evaluated in the present study, the results indicate that the aberrant expression of the TIC-snoRNAs is associated with aggressive biological behavior of NSCLC. Importantly, expression of the snoRNAs was inversely associated with overall survival of the patients. Therefore, the assessment of the TIC-snoRNA signatures might provide a potential approach for predicting outcome of NSCLC. However, as lung tumor is a heterogeneous disease, the analysis of the two TIC-snoRNA signatures cannot achieve the accuracy required to move forward for a clinical trial. Multiple biomarkers may provide more accuracy in prediction of outcome of lung cancer. From the identified 22 snoRNA signatures of lung TICs, we are optimizing a panel of biomarkers that can more precisely predict recurrence of NSCLC in our ongoing study.

snoRNAs have long been believed to modify and stabilize rRNAs for ribosome production. Several recent studies including our own observations demonstrate that dysregulation of snoRNAs is a common molecular event in carcinogenesis [14,15]. We previously found that snoRA42 activation promoted lung cancer development and progression [14]. The critic role of snoRA42 in TICs is first supported by that expression of snoRA42 is significantly elevated in CD133+ lung cancer cells compared with CD133- lung cancer cells. Furthermore, CD133- lung cancer cells with forced snoRA42 expression have the capacity to enhance the in vitro tumorigenicity, whereas the CD133- cells without forced snoRA42 expression do not. In addition, siRNA-mediated depletion of snoRA42 causes reduction of expression of stem cell-related genes, suppression of xenograft tumor formation, and inhibition of cellular proliferation and self-renewal of TICs in vitro. The inhibition of cell proliferation and self-renewal of lung TICs by snoRA42 dysregulation may be attained through inducing caspase 3-dependent apoptosis. Moreover, forced expression of snoRA42 in CD133- lung cancer cells produces higher cell proliferation and expression levels of stem cell-associated genes compared with CD133- lung cancer cells transduced with empty vector. Therefore, the present study extends the previous discoveries by finding that snoRA42 plays an important role in regulating stemness of lung TICs, and hence contributes to invasiveness and metastasis of NSCLC. Targeting snoRA42 in future may serve as an effective and specific therapeutic approach for lung cancer treatment. Nevertheless, investigating how the stem cell-core factors are regulated by snoRA42, and if other snoRNA signatures are also responsible to stemness features of TICs is warranted. In fact, we are functionally characterizing snoRA3 by using in vitro and in vivo approaches to determine if snoRA3 has a significant role in regulating stemness of lung TICs.

## Conclusions

We identified novel snoRNA signatures of lung TICs. Two of the signatures were evaluated in a cohort of NSCLC specimens. The results showed that the overexpression of the snoRNAs was inversely associated with survival of NSCLC patients. *In vitro* and *in vivo* functional analyses highlighted the importance of snoRA42 for the features of lung TICs. The snoRNA signatures of lung TICs may provide potential biomarkers for predicting outcome of NSCLC and novel therapeutic targets.

## Methods

### Patients and clinical specimens

To prepare single primary tumor cells, we obtained 28 lung tumor tissue specimens of NSCLC patents who underwent surgical resection in the University of Maryland Medical Center under appropriate institutional review board approval. All cases were diagnosed with histologically confirmed stage I NSCLC, comprising 15 patients with AC and 13 patients with SCC. Furthermore, to validate expression of snoRNAs in tumor tissue specimens, 82 frozen NSCLC tumor tissues were obtained. Clinical characteristics and histopathological data of the specimens are shown in Table 2. None of the patients had received preoperative adjuvant chemotherapy or radiotherapy before they received treatments.

**Table 2:** Demographic and clinical characteristics of 82 NSCLC patients

**Characteristics**	**No. of patients (%)**
Age at diagnosis	65.3 ± 9.5
Sex	
Male	53 (64.6)
Female	29 (3.45)
Race	
White	58 (70.7)
Black	19 (23.2)
Others	75 (6.1)
Smoker	
Yes	75 (91.5)
No	7 (8.5)
Tumor histology	
Squamous cell carcinoma	34 (41.5)
Adenocarcinoma	48 (58.5)
T stage	
I	27 (32.9)
II	28 (34.2)
III-IV	27 (32.9)

NSCLC, non-samll cell lung cancer.

### Tissue process and cell culture

The surgical tumor specimens were processed for preparing single dissociated cells as previously described [4]. Briefly, the surgical specimens were washed three times and left 12 hours in DMEM-F12 medium supplemented with penicillin/streptomycin and amphotericin B. Tissue dissociation was performed by adding enzymatic digestion with collagenase II (Gibco-Invitrogen, Carlsbad, CA) for two hours at 37°C. The dissociated cells were processed for flow cytometric isolation of TICs based on ALDH enzymatic activity as described below.

All NSCLC cell lines including A549, H299, H1792, H226, H522, H-1944, Calu-1, and H460 were purchased from the ATCC (Manassas, VA, USA). The cell lines were maintained as monolayer cultures in high glucose Dulbecco’s Modified Eagle Medium (DMEM) supplemented with 10% fetal bovine serum (FBS) and 50 μg/mL normocin (Invivogen, San Diego, CA) at 37°C in a humidified incubator with 5%.

### Flow cytometry isolation of ALDH1+ and ALDH1- cells

We isolated ALDH1 + cells from single dissociated cells of lung tumors using an Aldefluor assay kit (Aldagen Inc., Durham, NC) as described in our previous reports [5-7]. 1 × 10^6^ cells per well were suspended in Aldefluor assay buffer containing ALDH substrate, BAAA (1 μmol/L). Cells stained with 50 mmol/L of the specific ALDH inhibitor diethylaminobenzaldehyde (DEAB) were used as a negative control. Flow cytometric sorting was carried out using a FACSAria (Becton Dickinson, Mountain View, CA). Aldefluor fluorescence was excited at 488 nm, and fluorescence emission was detected using a fluorescein isothiocyanate (FITC) 530/30-nm band-pass filter by a FACS calibre machine (BD Biosciences, San Jose, CA).

### Flow cytometry isolation of CD133+ and CD133- cells from NSCLC cell lines

CD133+ and CD133- cells were isolated from NSCLC cell lines as previously described [4]. Briefly, cells were derived after trypsinization of adherent NSCLC cells. Cells were washed twice with magnetic automated cell sorting (MACS) buffer (Miltenyi Biotec Inc., San Diego, CA). Cell pellets were then resuspended in 300 μl of MACS buffer followed by addition of 100 μl of FcR blocking reagent (Miltenyi Biotec Inc.), 100 μl CD133 of microbeads (Miltenyi Biotec Inc.), and 50 μl of CD133/2-PE antibody (Miltenyi Biotec Inc.). The cells were washed with MACS buffer and MACS-assisted purification was performed according to manufacturer’s instructions. Purity of CD133+ cells and CD133- cells were checked by flowcytometry.

### TIC-conditioned suspension culture for sphere production

For sphere-forming cultures, cells were plated at a density of 2 × 10^4^ cells per well in six-well ultra-low attachment plates (Corning Inc., Corning, NY) in serum free Bronchial Epithelial Cell Basal Medium (BEBM), supplemented with the following growth supplements: BPE, Hydrocortisone, hEGF, Epinephrine, Transferrin, Insulin, Retinoic Acid, Triiodothyronine, and GA-1000, provided as BEGM prepackaged SingleQuots (Lonza, Walkersville, MD) plus rhEGF (Sigma, St Louis, MO), bFGF (Sigma). Fresh aliquots of EGF and bFGF were added every three days. Floating spheres were subjected to mechanical dissociation, followed by replating of single cells for propagation into a second, and subsequently into a third batch of suspension culture as described in our previous reports [5-7].

### RNA isolation

We used a mirVana miRNA Isolation Kit (Ambion, Austin, TX) to isolate RNA containing small RNA from cells and tissue specimens as described in our previous studies [24,25,15].

### snoRNA profiling of lung TICs

snoRNA profiling was performed by using GeneChipR Array (Affymetrix, Inc, Santa Clara, CA) as described in our previous report [15]. Microarray experiments were done with matched ALDH1+ and ALDH1- cells. One μg total RNA was labeled with Biotin FlashTag Biotin Labeling Kit (Affymetrix, Inc). The labeling reaction was hybridized on the arrays in Affymetrix Hybridization Oven 640 (Affymetrix, Inc) for 16 hours. The arrays were stained with Fluidics Station 450 using fluidics script FS450_0003 (Affymetrix, Inc), and then scanned on a microarray scanner (Axon Instruments Inc, Foster City, CA). snoRNA probe outliers were defined as per the manufacturer’s instructions (Affymetrix, Inc) and further analyzed for data summarization, normalization, and quality control by using the web-based QC Tool software (http://www.affymetrix.com). The normalized microarray data underwent further analysis as described in our previous study [15]. We performed tree visualization by using Java Treeview 1.0 (Stanford University School of Medicine, Stanford, CA).

### Quantification of snoRNA expression by qRT-PCR

Expressions of snoRNAs were determined by using real-time SYBR green RT-qPCR assay as described in our previous studies [14,15]. All PCR reactions were run on a CFX96 Real-Time qPCR detection system (Bio-Rad, Hercules, CA) using Brilliant SYBR green qPCR master mix (Agilent Technologies, Chelmsford, MA). U6 was used as an internal control gene. ΔCt was calculated by subtracting the Ct values of U6 from those of the snoRNA tested, and fold-change of each snoRNA was determined by the equation 2–ΔΔCt. qRT-PCR was also performed to decide expression levels of stem cell-associated genes, which included *ACTBF, ABCB1, ABCG2, CD133, Nanog, Nestin, Notch, Oct4, Smo, and Sox2*. The primer sequences are included in Additional file 1: Table S3.

### RNA interference assays

RNA interference assays were performed as described in our previous study [14]. Briefly, specific target site of designed siRNA in RNA sequence of snoRA42 (NCBI accession number NR_002974) was a 19 nucleotide stretch as follows: 5′-GTACCCATGCCATAGCAAA-3′. Corresponding non-targeting scrambled sequence was also designed. Transfection was performed by using Opti-MEM and Lipofectamine™ RNAiMAX Reagent (Invitrogen, CA, USA) as described elsewhere [26].

### snoRA42 overexpression vector construction and transduction with the vector

pCMV (pSilencer CMV 4.1; Ambion, Carlsbad, CA)-snoRA42 vector was constructed for ectopic snoRA42 expression as described previously [14]. A vector comprising sequence that showed no significant homology to the human genome databases was used as a control. Transduction of cells with the vectors was performed as described in our previous study [14].

### Methylthiazol tetrazolium (MTT), 5-bromo-2'-deoxyuridine (BrdU) incorporation, colony formation, transmigration, and wound healing assays

MTT and BrdU incorporation assays were performed as previously described [24,14]. Cells were seeded in 96-well plates at 1 × 10^4^ cells per well. Colony formation was determined by using 3D soft gar matrix with a 0.8% of soft agar (noble agar) base layer followed by 0.4% top agar layer containing cells at a density of 1 × 10^4^ with different treatments in 24-well culture plates. Colonies were counted using inverted light microscopy. For transmigration assay, the ability of migration and invasion of cells was examined using BD BioCoat Matrigel Invasion Chambers (BD Biosciences) according to manufacturer’s instruction. After 24 h, the migrated cells were pelleted and counted using a hematocytometer. To further confirm cell migration, a wound healing assay was performed in 12 well plates (1 × 10^5^ per well). When the cells were grown to 90 to 95% confluences, tranfection of CD133+ cells with siRNA-snoRA42 and scrambled siRNA, as well as mock transfection were performed. Wound lines were created manually by scratching the monolayer with a sterile 200 ml pipette tip and migration of the cells was assessed after 24 hours. Pictures were taken using Nikon inverted phase-contrast microscope (Nikon, Melville, NY). The distance between the parallel lines was measured using ImageJ software. All experiments were carried out at least three times.

### Apoptosis assay

Cell apoptosis was determined by two-color immunofluorescence staining followed by flowcytometric analysis. Cells were trypsinized without EDTA (Sigma) followed by two washing steps with phosphate buffered saline. 2 × 10^5^ cells per well were then incubated with 5 μl of FITC Annexin V (BD Pharmingen) and 5 μl of propidium iodide (BD Pharmingen) in 400 μl of 1 × binding buffer for 15 min at room temperature. Data acquisition and analysis were performed on a FACScan flowcytometer (Becton-Dickinson) using BD analysis software as described previously [14].

### Cleaved caspase ELISA

Cleaved caspase 3 ELISA was performed by using PathScan Sandwich ELISA kit (Cell signaling Technology, Inc., MA) according to manufacturer’s instructions. Cytochrome c-treated jurkat cell lysate was used as cleave caspase 3 positive control, whereas untreated jurkat cell lysate was used as cleaved caspase 3 negative control (Cell signaling Technology, Inc.). Cleaved caspase index was calculated as follows: (sample O.D. - negative control O.D./positive control O.D.-negative control O.D.)/100.

### Tumorigenicity assays in vivo

The animal study protocol was performed in accordance with the guidelines of University of Maryland School of Medicine Institutional Animal Care and Use Committee. Six 6-week old female SCID/Beige mice per group were subcutaneously inoculated with 5 × 10^5^ Calu-1-derived CD133+ cells with different treatments. The mice were observed for six weeks and then euthanized with CO_2_. Tumor volume was calculated by using the formula: width × width × length × 0.52 [5-7,14].

### Statistical analysis

Both univariate and multivariate Cox proportional hazard models were applied to assess the effect of various clinically and histopathologically interesting variants, and snoRNA expression on overall survival data of patients. Association of expression of the snoRNAs with overall survival rate was also analyzed using the Kaplan-Meier method. All P-values were determined by two-sided tests.

## Competing interests

The authors declare that they have no competing interests.

## Authors’ contributions

FJ designed the study, performed research, analyzed data and wrote the paper. KM, JL, and JS performed microarray and RT-qPCR, in vitro and in vivo analyses. ZL analyzed data. All authors have read and approved the final manuscript.

## Supplementary Material

Additional file 1 **Table S1.** Univariate Cox Proportional Hazards regression analysis of covariates in relation to survival of patients. **Table S2.** Multivariate Cox proportional hazards regression analysis to evaluate the independent prognostic value of snoRNA signature and clinical parameters. **Table S3.** Primer sequences of genes tested. Click here for file
