# Supplementary material for: Small nucleolar RNA signatures of lung tumor-initiating cells
Source: Mol Cancer. 2014 May 6;13:104. doi: 10.1186/1476-4598-13-104 (PMC4029979; doi:10.1186/1476-4598-13-104)
Supplement: Additional file 1 — Table S1. Univariate Cox Proportional Hazards regression analysis of covariates in relation to survival of patients. Table S2. Multivariate Cox proportional hazards regression analysis to evaluate the independent prognostic value of snoRNA signature and clinical parameters. Table S3. Primer sequences of genes tested. [file 1476-4598-13-104-S1.pdf]

Additional Table 1.

Univariate Cox Proportional Hazards regression analysis of covariates in relation to survival of patients

Covariate	Overall survival
Age	0.029
Sex	0.636
Smoking Status	0.394
Tumor histology	0.582
Stage	0.038
snoRA3 overexpression	0.002
snoRA42 overexpression	0.001

The numbers in the table represent P values calculated with the Wald test. P values <0.05 were considered statistically significant.

Additional Table 2.

Multivariate Cox proportional hazards regression analysis to evaluate the independent prognostic value of snoRNA signature and clinical parameters

Covariate	Overall survival
Age	0.026
Sex	0.625
Smoking Status	0.401
Tumor histology	0.634
Stage	0.043
snoRA3 overexpression	0.003
snoRA42 overexpression	0.002

The numbers in the table represent P values calculated with the Wald test. P values <0.05 were considered statistically significant.

Additional Table 3.

Primer sequences of genes tested

ACTBF: 5′-AGCCTCGCCTTTGCCGA-3′, ACTBR: 5′-GCGCGGCGATATCATCAT-3′
SnoRA42F: 5′-TGGTAATGGATTTATGGTGGGT-3′, SnoRA42R: 5′-GGACTGGGCAATGGTTCG-3′
U6F: 5′-CTCGCTTCGGCAGCACA-3′, U6: 5′-AACGCTTCACGAATTTGCGT -3′
ABCB1F: 5′-AAATTGGCTTGACAAGTTGTA TATGG-3′, ABCB1R: 5′-CACCAGCATCATGAGAGGAAGTC-3′
ABCG2F: 5′-TCA TCAGCCTCGATATTCCATCT -3′, ABCG2R: 5′-GGCCCGTGGAACATAAGTCTT-3′
CD133F: 5′-TGGATGCAGAACTTGACAACGT-3′, CD133R: 5′-ATACCTGCTACGACAGTCGTGGT-3′
NanogF: 5′-AATACC TCAGCC TCCAGCAGATG -3′, NanogR: 5′-TGCGTCACACCATTG CTATTCTTC-3′;
NestinF: 5′-CTGCGGGCTACTGAAAAGTT-3′, NestinR: 5′-AGGCTGAGGGACATCTTGAG-3′
Notch1F: 5′-CCTGAGGGCTTCAAAGTGTC-3′,Notch1R: 5′-CGGAACTTCTTG GTCTCCAG-3′
Oct4F: 5′-CTTGCTGCAGAAGTGGGTGGAGGAA-3′, Oct4R: 5′-CTGCAGTGTGGGTTTCGGGCA-3′
SmoF: 5′-TGGTCACTCCCCTTTGTCCTCAC-3′, SmoR: 5′-GCACGGTATCGGTAGTTCTTGTAGC-3′
Sox2F: 5′-AAATGGGAG GGGTGCAAAAGAGGA G-3′, Sox2R: 5′-CAGCTGTCATTTGCTGTGGGTGATG-3′.
